# The Persistence of *Staphylococcus aureus* in Pressure Ulcers: A Colonising Role

**DOI:** 10.3390/genes12121883

**Published:** 2021-11-25

**Authors:** Martin Fayolle, Madjid Morsli, Anthony Gelis, Marion Chateauraynaud, Alex Yahiaoui-Martinez, Albert Sotto, Jean-Philippe Lavigne, Catherine Dunyach-Remy

**Affiliations:** 1 Virulence Bactérienne et Infections Chroniques, INSERM U1047, Université de Montpellier, Service de Microbiologie et Hygiène Hospitalière, CHU Nîmes, 30908 Nîmes, France; fayolle.martin@gmail.com (M.F.); alex.yahiaoumartinez@chu-nimes.fr (A.Y.-M.); catherine.remy@chu-nimes.fr (C.D.-R.); 2 IRD, Microbes, Evolution, Phylogeny and Infection (MEPHI), Aix-Marseille-Université, IHU Méditerranée Infection, 13005 Marseille, France; mor_madjid@hotmail.com; 3 Centre Mutualiste Neurologique Propara, 34090 Montpellier, France; A.GELIS@propara.fr; 4 Virulence Bactérienne et Infections Chroniques, INSERM U1047, Université de Montpellier, 30908 Nîmes, France; marion.chateau@hotmail.fr; 5 Virulence Bactérienne et Infections Chroniques, INSERM U1047, Université de Montpellier, Service de Maladies Infectieuses et Tropicales, CHU Nîmes, 30908 Nîmes, France; albert.sotto@chu-nimes.fr

**Keywords:** chronic wound, colonisation, decubitus pressure ulcers, persistence, *Staphylococcus aureus*

## Abstract

Decubitus pressure ulcers (PU) are a major complication of immobilised patients. *Staphylococcus aureus* is one of the most frequently detected microorganisms in PU samples; however, its persistence and role in the evolution of these wounds is unknown. In this study, we analysed *S. aureus* strains isolated from PU biopsies at inclusion and day 28. Eleven *S. aureus* (21.1%) were detected in 52 patients at inclusion. Only six PUs (11.5%) continued to harbour this bacterium at day 28. Using a whole genome sequencing approach (Miseq^®^, Illumina), we confirmed that these six *S. aureus* samples isolated at D28 were the same strain as that isolated at inclusion, with less than 83 bp difference. Phenotypical studies evaluating the growth profiles (Infinite M Mano, Tecan^®^) and biofilm formation (Biofilm Ring Test^®^) did not detect any significant difference in the fitness of the pairs of *S. aureus*. However, using the *Caenorhabditis elegans* killing assay, a clear decrease of virulence was observed between strains isolated at D28 compared with those isolated at inclusion, regardless of the clinical evolution of the PU. Moreover, all strains at inclusion were less virulent than a control *S. aureus* strain, i.e., NSA739. An analysis of polymicrobial communities of PU (by metabarcoding approach), in which *S. aureus* persisted, demonstrated no impact of *Staphylococcus* genus on PU evolution. Our study suggested that *S. aureus* presented a colonising profile on PU with no influence on wound evolution.

## 1. Introduction

Pressure ulcers (PU) are a major complication of immobilised patients and, more particularly, of spinal cord injury (SCI) patients. PU annual incidence is estimated at 26% in this population, and more than 85% of SCI patients will develop at least one PU in their lifetime [1]. PU result from pressure phenomena associated or not with shear forces, and are classified into four clinical stages with two additional stages [2].

In SCI patients, PU are a major public health issue because they increase health care costs by a factor of four and the length of hospitalisation by a factor of six [3]. The occurrence of PU depends on factors related to care (e.g., adapted management of the disability, mobilisation), and intrinsic human factors (e.g., immune status, PU localisation). The wound microbiota also play a crucial role in delayed wound healing, and are represented as a polymicrobial community present in biofilm structure [4,5]. In this dynamic biofilm, bacteria adapt their virulence to persist in a colonising or infecting status.

According to the latest EPUAP/NPIAP/PPPIA (European Pressure Ulcer Advisory Panel/National Pressure Injury Advisory Panel/Pan Pacific Pressure Injury Alliance 2019) diagnosis recommendations of acute PU infection, it is strongly recommended that acute local and systemic signs of infection (without established scientific evidence) are detected. In these updated recommendations, bacterial bioburden is no longer listed as a criterion of PU infection [6]. Indeed, the presence of bacteria and its bacterial load is not sufficient for the diagnosis of wound infection. High diversity and bioburden of bacteria may be present in the wound without clinical signs of infection, making it difficult to distinguish colonisation and infection [7,8]. It is therefore essential to study host immune defences, bacterial virulence factors, as well as interactions between commensal and/or pathogenic microorganisms [4,9,10].

Among these polymicrobial chronic wounds, *S. aureus* is one of the frequently isolated bacteria [11,12]. *S. aureus* is a common coloniser of human epithelia, particularly the nose, but it is also present in both colonised and infected PU. It possesses a range of virulence factors aiding it to cause infections ranging from PU infection to oste(-omyel)itis and bacteraemia. Previous works have shown that the presence of *S. aureus* with high virulence potential is more significantly associated with severe grades of diabetic foot infection, and that some non- or low- virulent strains remain adapted to colonising chronic wounds [13,14]. *S. aureus* produces a large panel of virulence factors acting in bacterial adhesion and tissue colonisation, invasion of host cells and tissues, evasion of immune responses and biofilm formation [15,16,17,18,19,20].

Recently, we conducted a study (ESCAFLOR project) on the evolution of the wound microbiota over 28 days in SCI patients with pelvic PU [21]. Here, we evaluated the persistence and evolution of the virulence traits of *S. aureus* over time in these PU, and correlated this persistence with the clinical evolution and the polymicrobial environment of the chronic wound. The final aim was to characterise the role of *S. aureus* in PU evolution.

## 2. Materials and Methods

### 2.1. Bacterial Strains

All *S. aureus* strains were isolated during a prospective, monocentric clinical study (ESCAFLOR project) performed between May 2015 and September 2017 at the Centre Mutualiste Neurologique Propara (Montpellier, France). In this study, deep (biopsies) decubitus PU samples were collected from 55 patients at inclusion (D0) and 28 days after (D28). Biopsies were immediately cultured at the Department of Microbiology at Nîmes University Hospital (France). A total of 52 couples of biopsies was analysed. Bacterial identification was obtained by mass spectrometry (Vitek-MS^®^, Biomérieux, Marcy-l’Étoile, France) and antibiograms were performed by the disc diffusion test according to the European Committee for Antimicrobial Susceptibility Testing (EUCAST) recommendations [22].

Qualitative (RYB (Red-Yellow-Black) wound classification) and quantitative (maximal length and maximal perpendicular width, depth using a ruler) criteria were used to classify more precisely wounds evolution into “Improved” and “Worsening” groups against EPUAP criteria. We used these criteria to evaluate the impact of the presence of *S. aureus* in the evolution of the PUs.

The low virulent *Escherichia coli* OP50 strain (corresponding to the nutrient of nematodes) and a virulent *S. aureus* NSA739 strain (isolated from Grade 3 infected DFU and belonging to our collection) were used as control in *Caenorhabditis elegans* experiments.

### 2.2. Whole Genome Sequencing

DNA extraction was performed with DNeasy UltraClean Microbial Kit (Qiagen, Hilden, Germany) according to the manufacturer’s instructions. Twelve *S. aureus* strains were sequenced by Illumina MiSeq Sequencing system (Illumina, San Diego, CA, USA), with Nextera XT DNA Library Prep Kit (paired-end read libraries, Illumina) according to supplier’s recommendations. Quality control of the reads was performed with FastQC software (v.0.11.7). SPAdes software was used for genome assemblies [23]. Bacterial genome annotation was performed with Prokka [24]. MLST, SpA Typing, ResFinder 4.1, VirulenceFinder-2.0 and PlasmidFinder 2.1 were used for sequences analysis [25,26,27,28] (https://cge.cbs.dtu.dk (accessed on 19 May 2021)). The contigs were matched against the NBCI database to obtain the closest *S. aureus* reference sequence for the 12 strains isolated from the wounds at both D0 and 28 (Table S1). The reads were aligned to reference sequences using the CLC Genomics Workbench software (Qiagen, Germantown, MA, USA). To determine the difference of genes and Single Nucleotide Polymorphisms (SNPs) of our strains between D0 and D28, Roary [29] and Snippy [30] software were used online (usegalaxy.org (accessed on 21 May 2021)). Genome-wide representation was performed with phandango application [31]. Whole Genome bioinformatic data are summarised in Table S1.

### 2.3. In Vivo Caenorhabditis elegans Killing Model

The nematode infection assay was carried out as previously described using the Fer-15 mutant line worms, fertile at 15 °C and sterile at 25 °C [32,33]. Fer-15 was provided by the Caenorhabditis Genetics Center, which is funded by the NIH National Center for Research Resources (NCRR). Nematodes were first synchronised at the same development stage using bleach (hypochlorite method). Overnight cultures of controls (*E. coli* OP50, and NSA739), and the 12 studied *S. aureus* strains in nematode growth medium (NGM) were harvested, centrifuged, washed once, and suspended in phosphate buffered saline solution (PBS) at pH 7.0 at a concentration of 10^5^ CFU/mL. NGM agar plates were inoculated with 10 µL of bacterial suspension and incubated at 37 °C for 8–10 h. Plates were brought back to room temperature and seeded with L4 stage worms (approx. 40 per plate). Plates were then incubated at 25 °C. The worm survival rate was assessed daily with a stereomicroscope (Nikon SMZ 745, Amstelveen, The Netherlands). A nematode was considered dead when it no longer responded to touch. *C. elegans* that died from being trapped by the wall of the plate were excluded from the analysis. These experiments determined the lethal time 50% (LT50), which corresponded to time (in days) required to kill 50% of the initial worm population. All experiments were performed in biological triplicate, repeated twice for each selected strain.

### 2.4. Growth Curves Evaluation

Growth profiles of the 12 *S. aureus* strains were performed in Luria Broth (LB) medium. Briefly, bacterial suspensions were calibrated to obtain an optical density (at 600 nm) around 0.1. Each bacterial suspension was added to a 48-well plate. The plates were incubated at 37 °C for 48 h and the absorbance at 600 nm of each well was determined with the Infinite M Mano automatic absorbance reader (Tecan, Männedorf, Switzerland). We used the nonlinear regression model of Gompertz [34] to obtain the equations of each growth curve, as well as various notable points (Ym, Y0, K and 1/K), with GraphPadPrism 9.1.0 software (San Diego, CA, USA). The Gompertz equation can be written as follows:

$$Y\left(t\right)=\mathrm{Ym}*({\left(\frac{Y0}{\mathrm{Ym}}\right)}^{\exp\left(-K*t\right)})$$

where Y(t) corresponds to the absorbance at a time t, Ym corresponds to the maximum absorbance (stationary phase), Y0 corresponds to the absorbance at t = 0 h, K determines the lag time (1/h) and 1/K (h) the inflection point of the exponential phase. Following the inflection points, we were able to determine the beginning of the exponential phase, and thus, to calculate the slope of the linear trend line of this phase. This model has been validated in different research areas [35] and correlated strongly with our raw data (R^2^ equal to 0.99 for P5 D0/D28; 0.99 for P7 D0; 0.98 for P7 D28; 0.99 for P32 D0/D28; 0.99 for P37 D0/D28; 0.99 for P41 D0; 0.98 for P41 D28; 0.98 for P50 D0/D28). Each experiment was performed in duplicate.

### 2.5. Determination of Early Biofilm Formation

To explore capacity of *S. aureus* to form biofilm, we used the Biofilm Ring Test^®^ technique (Biofilm Control, St. Bauzire, France) following the manufacturer’s recommendations [36]. Standardised bacterial cultures were deposited on 96-well plates containing magnetic beads. After incubation at 37 °C, the plates were inserted onto a magnetic block and then into a reader (Epson Scanner modified for microplate reading). Images of each well were acquired at 1.5 h, 2.5 h, 3.5 h, before and after magnetisation. Data were analysed by the BFC Elements 2.0 software (Biofilm Control) giving a result in the form of a Biofilm Formation Index (BFI). A BFI > 7 corresponds to a complete mobility of magnetic beads and thus an absence of biofilm. A BFI < 2 indicates an immobility of beads, blocked by the formed biofilm. A BFI between 2 and 7 corresponds to a biofilm in formation [37]. Three experiments with two repeats were performed in Brain Heart Infusion (BHI) medium.

### 2.6. Description of Bacterial Communities Associated with S. aureus Using Metabarcoding Approach

After digestion with proteinase K at 56 °C for 3 h, bacterial DNA was extracted from biopsies obtained at D0 and D28. Tissue samples were lysed using MagNA Lyser Instrument^®^ (Roche, Meylan, France). A 300 µL sample was added into prefilled disposable vials containing ceramic bead compatible with MagNA Lyser and centrifuged twice at 5000× *g* rpm for 60 s. Samples were centrifuged briefly and DNA was extracted from 200 µL of the supernatant using the EZ1 DNA Tissue kit (Qiagen, Courtaboeuf, France) according to the manufacturer’s instructions. DNA was eluted with 100 μL ultrapure Molecular Biology grade water. An extraction control with ultrapure Molecular Biology grade water was used. The concentration of extracted DNA was measured by spectrophotometry (Nanodrop^®^, ThermoScientific, Illkirch, France).

The bacterial communities of the gDNA samples were analysed with a metabarcoding approach based on a process developed, optimised and standardised by GenoScreen (Lille, France). First, amplicon libraries were prepared according to the Metabiote^®^ solution. Extraction controls (PCR-quality water having undergone the same extraction process) were also performed. Libraries were generated targeting the V3-V4 region of the 16S rDNA. The sequencing of the amplicon libraries was performed on a single run Miseq (Illumina^®^, Paris, France) “paired-end” in 2 × 250 base chemistry. The “merging” step or assembly of the “paired-end” readings was carried out using the FLASH tool [38] applying a 97% nucleic identity assembly over the entire overlap area. Similar sequences were clustered at a defined identity threshold (97% identity for genus affiliation on the targeted 16S rDNA region) with Uclust v1.2.22q [39,40]. An OTU (Operational Taxonomic Unit) table was generated and was expressed in RA (%).

### 2.7. Statistical Analysis

Statistics were performed with GraphPad Prism 9.1.0 (San Diego, CA, USA) and graphs were created online with BioRender.com. A statistically significant difference is retained for *p* < 0.05. Wilcoxon Mann Withney Test was performed to compare exponential phase of the different *S. aureus* growth profiles. The LT50 of worms fed with the different pairs of *S. aureus* involved in improved or worsening evolution of the wounds were compared using the Wilcoxon Mann Whitney Test. Differences in survival rates for each strain were analysed by a Mantel-Cox test. BFI values between D0 and D28 (for each strain), and following the clinical evolution of the PU (at D0 and D28) were tested using the Mann Whitney Test. *Staphylococcus* RA at D0 and D28 (for each strain) and following the clinical evolution of the wounds (at D0 and D28) were also tested using the Mann Whitney Test.

## 3. Results

### 3.1. Description of Patients

Samples from 55 patients were initially included in this study (Figure S1). Finally, 52 PU biopsies collected on D0 and D28 were analysed. *S. aureus* was isolated in 11 biopsies (21.1%) at D0 and in six biopsies (11.5%) at both D0 and D28. These six patients were included in this study. Three presented PU with improved evolution (P5, P32, P41), while three had worsening evolution of their wounds (P7, P37, P50) at D28, despite appropriate management. Demographic, clinical and biological data of patients with *S. aureus* strains isolated on PU at D0 et D28 are presented in Table 1**.**

### 3.2. Genotypical Characteristics of S. aureus Strains

#### 3.2.1. Genotyping of *S. aureus* Strains

The 12 *S. aureus* were sequenced to determine the genome evolution between D0 and D28, and to compare genome composition between strains isolated from wounds with improvement versus strains isolated from worsening wounds at D28 (Table S1). All pairs of strains harboured the same Sequence Type (ST) at D0 and D28. If we follow the criteria proposed by Ankrum et al. (who defined two strains as identical if the number of SNPs was less than 71) [41], all strains except P50 can be considered identical between D0 and D28 (Table 2). Concerning the P50 strains, a difference of 82 SNPs was observed between D0 and D28, showing that these strains were very closely related (<123 SNPs following the Ankrum et al. classification [41]). In wounds with an improvement, ST398 was detected in 2/3 *S. aureus* pairs and ST8 in the remaining pair. In wounds with worsening evolution, ST5 was the most detected in 2/3 *S. aureus* pairs. Genome-wide representation confirmed that the pairs of strains were the same between D0 and D28, showing that in all cases, the same *S. aureus* had persisted in the six PUs for 28 days (Figure S2).

#### 3.2.2. Resistome and Virulome of *S. aureus* Strains

P7 and P37 strains harboured a *mecA* gene at inclusion and D28, showing that methicillin-resistant *S. aureus* (MRSA) strains were only isolated in the worsening wounds (Table 3). P7 genomes also contained a mutation in *gyrA* (corresponding to p.S84L in gyrase A protein) and *glrA* (corresponding to p.S80Y in topoisomerase IV subnit A protein) genes. P37 genomes harboured two mutations in *glrA* gene (p.S80F; p.E84K) and one mutation in *gyrA* (p.S84L). Among the MSSA strains, P32 D0 had mutations in *fusA* (corresponding to p.L461S conferring acid fusidic resistance), and *glrA* (p.E84G) genes. Interestingly, mutation in *glrA* gene was absent in the P32 strain isolated at D28 and has been confirmed phenotypically with a susceptibility to fluoroquinolones. Finally, *ermA* gene (conferring resistance to erythromycin and clindamycin) was only detected in the pair of P41 genomes.

The panel of the main virulence factors-encoding genes was conserved in all pairs of *S. aureus* strains between D0 and D28 (Table 3). In stagnating wounds, two MRSA strains (P7 and P37) had additional genes: *sed, sej* and *ser* contained in a plasmid (SAP060A). Finally, P37 and P50 harboured an enterotoxin gene cluster (with the presence of *seg*, *sel-i*, *sel-m, sel-n, sel-o, sel-u*, and *sel-p*).

#### 3.2.3. Evaluation of *S. aureus* Virulence Strains according to Clinical Evolution

To understand the virulence potential of the pairs of *S. aureus* isolated at inclusion and D28 according to clinical evolution of the PU, the *C. elegans* in vivo model was used. Data were compared to the results obtained with OP50 (a low virulent *E. coli* strain) and NSA739 (a virulent *S. aureus* strain isolated from diabetic foot infection). All *S. aureus* strains killed the nematodes more rapidly than the avirulent *E. coli* OP50 strain used as nutrient for the worms (*p* < 0.001) (Table 4). One strain (P5) isolated at inclusion presented no significant virulence with NSA739, although its LT50 increased (LT50: 3.3 days ± 0.6 vs. 3.0 ± 0.1, respectively) (Table 4). The other strains showed significantly decreased virulence compared with NSA739 (LT50: 3.7 to 4.8 days ± 0.7 vs. 3.0 ± 0.1, respectively; *p* < 0.01). No link with the evolution of the PU was observed (Table 4).

When the *S. aureus* strains isolated at D0 were compared to the clonal strains isolated at D28, their virulence was significantly decreased at D28 for P5, P7, P32, P37 and P41 strains (LT50: 3.3 to 4 days ± 0.7 vs. 3.7 to 4.7 ± 0.7, respectively; *p* < 0.01). No significant difference of LT50 was noted for the P50 strain, irrespective of the time of isolation (D0 vs. D28), although an increased nematode lifespan was noted: 4.6 days ± 0.2 vs. 5.0 ± 0.1 (*p* = not significant) (Table 4). Finally, the virulence profiles of the strains isolated at D0 and D28 were not correlated with wound evolution. Indeed, LT50s obtained from *S. aureus* strains isolated in the wounds with an improvement were similar from those obtained from strains isolated in the worsening wounds (*p* = 1 and 0.64, for the comparison of LT50s at D0 and D28, respectively).

### 3.3. Evolution of S. aureus Fitness and Ability to Form Biofilm in Accordance to Clinical Evolution

The fitness and capacity of strains to form biofilms was studied to describe the phenotypic characteristics of *S. aureus* persisting in PU, and to establish whether persistence was associated with improvement (P5, P32, P41) or worsening (P7, P37, P50) of the wounds.

#### 3.3.1. Fitness of *S. aureus* Strains

**Figure 1:**
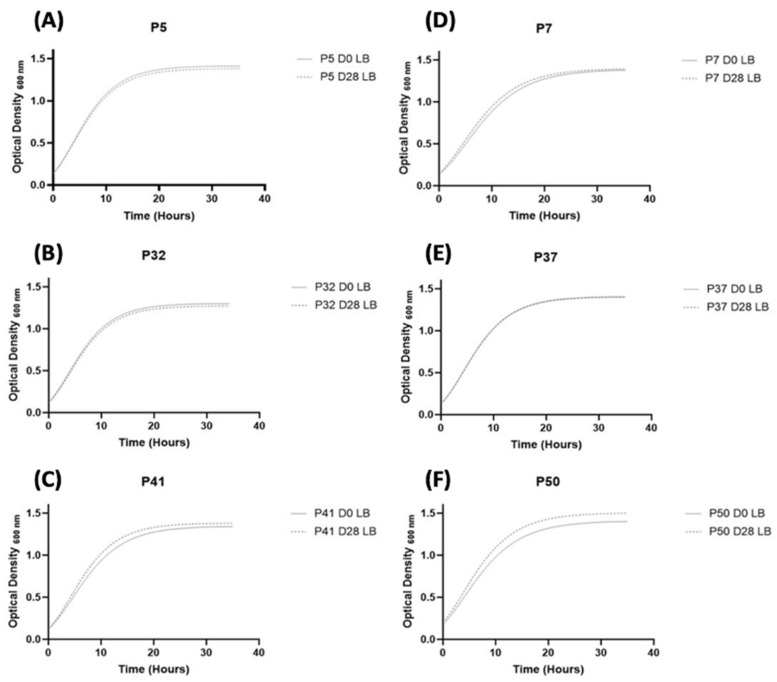
Growth curves of *S. aureus* strains using the Gompertz equation. P5 (**A**), P32 (**B**), P41 (**C**) belong to the wounds with an improving evolution. P7 (**D**), P37 (**E**), P50 (**F**) belong to the worsening wounds.

The growth profiles of each strain were compared by monitoring absorbance over time. No significant differences between the pairs of strains isolated at D0 and D28 were observed for the maximum absorbances in stationary phase (Ym), except for P50. The growth of P50 strain at D28 was significantly faster and more pronounced than that of P50 isolated at inclusion (1.506 and 1.409 respectively, disjoint interval confidence) (Figure 1). The comparison of Ym between each *S. aureus* strains showed that only one strain, P32 D0/D28 (isolated from a wound with a clinical improvement), presented a lower Ym compared to the Ym of all strains isolated from worsening wounds (Ym, D0 = 1.302 and Ym D28 = 1.273, respectively, disjoint interval confidence). Lag times (1/K) and exponential growth phase between D0 and D28 were not significantly different for any strain (*p* = 1 in strains isolated from improving wounds and 0.7 in strains isolated from worsening wounds) (Table S2). Finally, no significant difference in slopes was observed between the strains, regardless of the clinical evolution either at D0 and D28 (*p* = 1 and 0.2, respectively), showing that bacterial fitness was independent of wound evolution.

#### 3.3.2. Ability to Form Biofilm in *S. aureus* Strains

**Figure 2:**
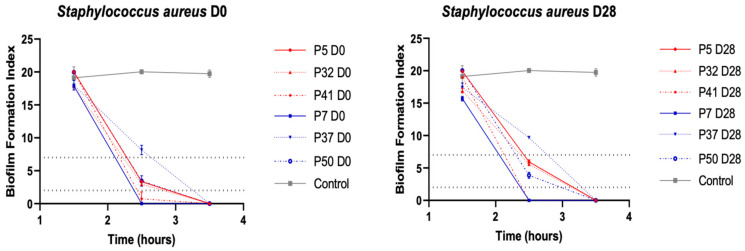
Biofilm formation of pairs of *S. aureus* isolated from pressure ulcers at D0 and D28. The kinetics of early phase of biofilm formation were determined on P5, P7, P32, P37, P41 and P50 by the BioFilm Ring Test^®^ (BioFilm Control, France). On right, study of *S. aureus* isolated at inclusion; on left, study of *S. aureus* isolated at day 28. In blue, *S. aureus* strains belonging to worsening wounds, in red, *S. aureus* belonging to improving wounds. Dotted horizontal lines: >7, no biofilm; <2, fixed biofilm, 2 < BFI < 7, biofilm in formation. Means ± standard errors of the mean of BFIs for at least three independent replicates are presented.

The ability of *S. aureus* strains to form biofilms was analysed by the Biofilm Ring Test^®^ (Biofilm Control, France). All strains were able to produce an early biofilm after 3.5 h of incubation (Figure 2). No significant difference in biofilm formation was observed between each pair of strains isolated at D0 and D28 (*p* between D0 and D28 = 0.66; 1; 0.46; 0.65; 0.89; 0.5 for P5, P32, P41, P7, P37, P50, respectively). However, some differences were noted: at 2.5 h of incubation, P37 D0 and D28 strains did not produce biofilm, whereas P7 and P41 produced a fixed biofilm. P5, P32 and P50 (D0 and D28) presented a preformed biofilm (2 > BFI < 7). No difference between strains was noted, regardless of the clinical evolution of the wounds from which they were isolated (*p* = 0.93 and 0.98, at D0 and D28, respectively).

Globally, no modification of *S. aureus* growth profile and ability to form biofilm was observed over time, irrespective of the PU clinical evolution.

### 3.4. S. aureus and Bacterial Interactions: Impact on Wound Evolution?

**Figure 3:**
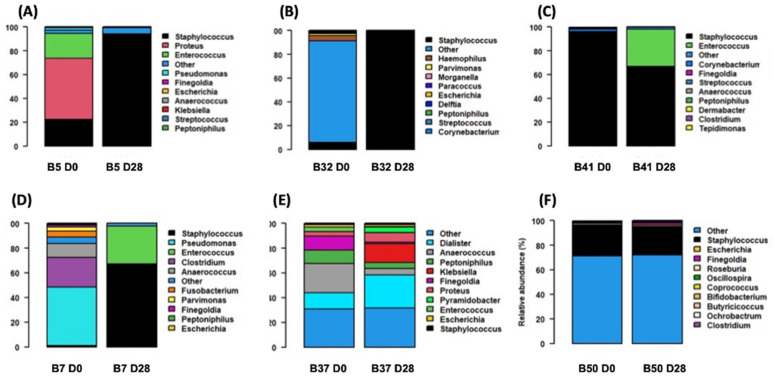
Relative abundance of the 12 top bacterial species from decubitus PU for each biopsy at D0 and D28. At the top, B5 (**A**), B32 (**B**), B41 (**C**) are biopsies sampled from PU that improved at D28. At the bottom, B7 (**D**), B37 (**E**) and B50 (**F**) are biopsies sampled from PU that worsened at D28.

Bacterial communities from PU biopsies where *S. aureus* were isolated were described using a metabarcoding approach over time (Figure 3). In wounds with an improvement, the PU biopsy performed on patient 5 (B5) showed that *Staphylococcus* genus relative abundance (RA) increased between D0 and D28 (+71.5%) inversely to *Enterococcus* and *Proteus* RA, which decreased strongly between D0 and D28 (−51.2% and −20.9%, respectively). For PU biopsy (B32), the same trend was noted, with an increase of *Staphylococcus* genus RA (+93.2%) between D0 and D28. In the last PU biopsy (B41), *Staphylococcus* RA was initially high at D0 at 95.3% and then decreased to 67.0% at D28, in favour of *Enterococcus* RA, which increased to 31.2% at D28.

In wounds with worsening evolution, the two PU biopsies (B7 and B37) had very low *Staphylococcus* genus RA at D0 (both 1.1%). Moreover, the B7 D0 wound microbiome was composed of a high diversity of bacterial genus with a majority of *Pseudomonas*, *Clostridium* and *Anaerococcus*. This diversity decreased at D28 in favour of *Staphylococcus* RA, which increased significantly (+66.9%). The B37 biopsy showed that *Staphylococcus* RA plateaued at 1.1% at D28. The microbiome remained highly diverse, with a persistence of *Dialister*, *Anaerococcus*, *Peptoniphilus* and *Proteus.* In the last PU biopsy (B50), *Staphylococcus* RA remained constant between D0 and D28 (25% and 23%, respectively).

Globally, *Staphylococcus* genus persisted between D0 and D28 with a higher RA at D28 in the wounds with a clinical improvement compared with the worsening wounds, but without significance (*p* = 0.7).

## 4. Discussion

The main objective of this study was to evaluate the persistence of *S. aureus* in PU using genotypic and phenotypic approaches, and the link between this persistence and the clinical evolution of the wounds, despite a similar and appropriate wound management (wound debridement, wound dressings, antibiotherapy according to antibiogram results etc.). To date, only one longitudinal study has evaluated the ability of this species to persist in chronic wounds [42]. Characterising persistence is crucial to understanding bacterial adaptation in this specific environment. Firstly, we observed that, although *S. aureus* is very common in DFU (35–60% of cases), its presence in PU is less frequent [13]. In our study, this species was isolated in only 23.4% of biopsies at D0. Moreover, only six (11.5%) patients presented a persistent colonisation by a related strain over 28 days, although this represents 54.6% of the patients harbouring *S. aureus* (6 cases/11). This persistence is lower than that observed in diabetic foot ulcers (DFU) (25%) [42], and confirmed a low implantation rate compared with other chronic conditions like lung colonisation in *Cystic Fibrosis* (CF) patients [43]. The dynamics of *S. aureus* implantation in PU are likely to differ slightly from those of bacterial colonisation and adaptation in the lower respiratory tract in CF due to distinct environmental conditions and infection management. However, as observed in CF, our results support that the presence of *S. aureus* in PU is relatively stable over the course of chronic wounds. In our study, the presence of *S. aureus* was associated with different wound evolutions: three PU showed clinical improvement, whereas three others had a worsening evolution. To gain insight into the adaptive ability of *S. aureus* in PU, we compared the pairs of strains with a genomic and phenotypic approach in order to better characterise their implication in the clinical evolution of the wounds.

Genomic analysis confirmed that the same strain persisted in the PU in all six cases. Indeed, the pairs of isolates belonged to the same ST and were considered as the same strain in five cases according to Ankrum et al. criteria (<71 SNPs differences), or very closely related in one case (P50) (<124 SNPs differences) [41]. No modification in genome size or acquisition/loss of virulence and resistance genes were observed between D0 and D28, except for P32, which lost its mutation in *grlA* gene at D28 (Table S1). This suggests that the bacterial adaptation in this hostile environment does not require the reduction or major modification of the genome, as previously noted [44]. Even if the length of our study was limited to 28 days, Uhlemann et al. observed that persistent strains of *S. aureus* in skin infections underwent limited genome evolution over a longer period (15 months) [45]. Preliminary tests were performed to investigate the genomic diversity of *S. aureus* in PU biopsies. Five isolates were selected from B5 biopsy cultures and MLST typing was performed. All five isolates had the same sequence type, suggesting that the isolates were the same. For the other samples, we selected only one *S. aureus* isolate per sample, and proposed that the different colonies belonged to a same clone. These strains could persist over time, regardless of wound stage, clinical evolution and associated ST. The ST described in our study are those frequently detected in other chronic wounds, such as DFU [46]. ST398 (MSSA) was isolated from 33.3% (2/6) of wounds harbouring *S. aureus*, and exclusively in wounds with a clinical improvement. The presence of this clone in our population has been previously described, and this clone became a predominant lineage in France [47]. MSSA-ST398 was frequently implicated in severe infections such as bloodstream infections, endocarditis and bone joint infections [46,47]. This suggests that this clone is able to adapt to the chronic wound environment and modify its virulence. Among *S. aureus* clonal lineages, MRSA was identified in 3.8% of the patients at inclusion (2/52) and 33.3% of the patients with a persistence of *S. aureus* during the study (2/6). This low prevalence must be underlined, especially as rehabilitation units experience high presence of multidrug resistant bacteria [48]. Although the two pairs of strains belonged to the most frequent Clonal Complexes (CC) isolated from chronic wounds (CC5-MRSA (Pediatric Clone) and CC8-MRSA (Lyon Clone)) and were associated with worsening wounds, their low representation and implantation rate in PU suggest that their virulence potential remains contentious.

Our study also demonstrates that *S. aureus* persistence in PU is not predictive of wound evolution or the development of an infection. This suggests that other elements must be taken into account to predict wound evolution. The genomic approach provided no clear explanation for the difference of clinical evolution of wounds infected by *S. aureus*. Experiments to understand the virulence potential of this bacterium isolated from PU were conducted, evaluating their fitness, biofilm formation capacity and virulence in a *C. elegans* in vivo model. We showed that the fitness and biofilm forming capacity of these strains did not change over time, regardless of the clinical evolution of the wounds. Uhlemann et al. also identified similar growth profiles between *S. aureus* strains that persistently colonised the skin [45]. Moreover, we observed that all strains presented decreased virulence profiles between D0 and D28 (although this decrease was not significant for P50), irrespective of the clinical evolution of the wounds. We could hypothesise that either the chronic wound environment or the polymicrobial environment influences the virulence of *S. aureus*, as previously demonstrated [49]. More interestingly, all *S. aureus* strains (isolated at D0) presented a lower virulence in the *C. elegans* model compared with our *S. aureus* virulent control NSA739 (Table 4). This result could suggest that *S. aureus* present in PU have modified virulence potential. Further work on the transcriptomic profile of these persistent isolates must be undertaken to evaluate the regulation of the different pathways involved in this evolution. Another explanation could be the influence of other bacteria present in the PU that could modulate the virulence of *S. aureus*. To explore this hypothesis, a specific microbiota analysis was performed following our first main study [21]. The cutaneous microbiota present in chronic wounds is particularly diverse, and bacteria are organised into pathogroups or functionally equivalent pathogroups, to form a bacterial community in the extracellular matrix [4]. Previous studies have observed that commensal bacteria (such as *Helcococcus kunzii* and *Corynebacterium striatum*) can influence the virulence of *S. aureus*, with a strong decrease of some important virulence genes (*hla*, *psm* and *agr*) and an increase of *spa* genes, resulting in greater adherence to epithelial cells and a shift in virulence to a commensal state [32,50]. We would expect an increase of the *spa* gene expression within the PU. Moreover, the persistence of *S. aureus* seems to be more related to the expression of the virulence genes, biofilm organisation and the environment (e.g., cutaneous and digestive microbiota, bacterial interaction) in which it is found rather than its genome composition, which is very stable among the various species [51]. In our study, *Staphylococcus* genus RA was particularly prevalent in the three wounds which showed clinical improvement, whereas this genus was less present in the worsening wounds. As *Staphylococcus* genus RA did not correspond exclusively to *S. aureus* but to all species of *Staphylococcus* sp., the data could indicate that a high diversity of *Staphylococcus* species could participate in the modulation of *S. aureus* virulence [52]. However, the situation seems to be more complex: *Staphylococcus haemolyticus* were detected in B5 D0 culture, *S. epidermidis* in B7 D0 and D28, B32 D28 and B41 D28. Moreover, *S. aureus* was the only representative of *Staphylococcus* genus at inclusion (B32 D0, B41 D0, B37 D0, B50 D0), at D28 (B5 D28, B37 D28, B50 D28), regardless of the clinical evolution of the wound (improvement: B5, B32, B41 vs. worsening evolution: B37, B50). While this result could indicate that the exclusive presence of *S. aureus* over 28 days in two biopsies (B37 and B50) was associated with a worsening evolution of the PU, this was not confirmed in the P5 patient, who showed wound clinical improvement with only the presence of *S. aureus* at D28. Altogether, our study suggests that the modulation of *S. aureus* virulence in PU results from multifactorial events, and it seems that *S. aureus* in PU acts more frequently as a coloniser than a pathogenic strain. Indeed, other bacteria species are more readily involved in the infection of decubitus PU, and therefore, in the clinical evolution of wounds, than *S. aureus.*

## 5. Conclusions

Even if a larger scale study should be conducted, our work highlights that *S. aureus* persistence is a rare event in PU. Moreover, this persistence is not linked to the clinical evolution of the wound, and probably depends on gene expression and the influence of bacterial cooperation on *S. aureus* virulence and biofilm organisation. The interaction between bacteria explored by the microbiota analysis and the effect of the environment encountered by bacteria inside the PU could explain why the virulence of *S. aureus* on PU undergoes modulation before infection, and acts more frequently as a coloniser than a pathogen. This work provides the basis for an understanding of *S. aureus* colonisation dynamics in chronic wounds.

## Figures and Tables

**Table 1:** Clinical characteristics of SCI patients with pressure ulcers infected or colonised by *S. aureus* after 28 days and according to wound evolution (improvement vs. worsening evolution).

Variables		Pressure Ulcers Evolution during 28 Days					
		Improvement (*n* = 3)			Worsening (*n* = 3)		
Patient ID		P5	P32	P41	P7	P37	P50
Sex		F	M	M	M	M	F
Age	Years	76	42	61	52	66	62
Wound stage * (at D0)		III	III	III	III	III	III
Wound stage (at D28)		III	III	III	III	III	III
Wound localisation	Ischial/Sacral	Sacral	Ischial	Sacral	Sacral	Ischial	Ischial
CRP (D0)	mg/L	26	22	11	9	154	5
CRP (D28)		23	34	10	22	69	9
Number of antibiotics used during wound management		1	0	0	0	1	0

* grade NPUAP (US National Pressure Ulcer Advisory panel), Male = M; Female = F; CRP = C-reactive protein. Wound stage III corresponds to full thickness tissue loss. Subcutaneous fat may be visible, but bone, tendon and muscle are not exposed.

**Table 2:** Genomic characterisation of *S. aureus* strains isolated from pressure ulcers at inclusion (D0) and day 28 (D28).

Wounds Evolution	Strains	Sequence Type Susceptibility to Methicillin ^1^	SpA Type	Difference in SNP ^2^ Numbers (D0/D28)
Improvement	P5 D0	398—MSSA	t3625	6
	P5 D28			
Improvement	P32 D0	398—MSSA	t571	43
	P32 D28			
Improvement	P41 D0	8—MSSA	t008	12
	P41 D28			
Worsening	P7 D0	8—MRSA	t008	32
	P7 D28			
Worsening	P37 D0	5—MRSA	t777	8
	P37 D0			
Worsening	P50 D0	5—MSSA	t002	82
	P50 D28			

^1^ MSSA: Methicillin susceptible *S. aureus*. MRSA: Methicillin resistant *S. aureus*; ^2^ SNP: single nucleotide polymorphisms.

**Table 3:** Main virulome and resistome traits of *S. aureus* strains isolated from pressure ulcers at inclusion (D0) and day 28 (D28).

Funtions	Genes	PU with Clinical Improvement ^3^						PU with Worsening Evolution ^3^					
		P5 D0	P5 D28	P32 D0	P32 D28	P41 D0	P41 D28	P7 D0	P7 D28	P37 D0	P37 D28	P50 D0	P50 D28
Adhesion/Colonisation	*fnbpA*	1	1	1	1	1	1	1	1	1	1	1	1
Biofilm Formation Regulatory System	*icaABCDR* ^1^	1	1	1	1	1	1	1	1	1	1	1	1
	*agrA, agrB* *sarA*	1 1	1 1	1 1	1 1	1 1	1 1	1 1	1 1	1 1	1 1	1 1	1 1
Pore Forming Toxins	*lukDE*	0	0	0	0	1	1	1	1	1	1	1	1
	*hlgA, hlgB, hlgC*	1	1	1	1	1	1	1	1	1	1	1	1
	*hly*	1	1	1	1	1	1	1	1	1	1	1	1
	*psm*	0	0	0	0	0	0	0	0	0	0	0	0
	*lukF-PV, lukS-PV*	0	0	0	0	0	0	0	0	0	0	0	0
Toxins that induce Lymphocyte T activation	*sea and sel-X*	1	1	1	1	1	1	1	1	1	1	1	1
	*sed, sej, ser*	0	0	0	0	0	0	1	1	1	1	0	0
	*seg, sel-i, sel-m, sel-n, sel-o, sel-u, sel-p*	0	0	0	0	0	0	0	0	1	1	1	1
	*tsst-1*	0	0	0	0	0	0	0	0	0	0	0	0
Avoid Host Immune Response	*clfB*	0	0	0	0	1	1	1	1	1	1	1	1
	*coA*	1	1	1	1	1	1	1	1	1	1	1	1
	*clfA*	0	0	0	0	1	1	1	1	1	1	0	0
	*spA*	1	1	1	1	1	1	1	1	1	1	1	1
Protease activity	*aur*	1	1	1	1	1	1	1	1	1	1	1	1
	*etA, etB, etD*	0	0	0	0	0	0	0	0	0	0	0	0
	*splA, splB, splE*	0	0	1	1	1	1	1	1	1	1	1	1
Other	*sak*	0	0	0	0	0	0	0	0	1	1	1	1
Resistance to β-lactams (MRSA ^2^)	*mecA*	0	0	0	0	0	0	1	1	1	1	0	0
Resistance to quinolones	*glrA and/or gyrA mutations*	0	0	1	0	1	1	1	1	1	1	0	0
Resistance to macrolides and related	*ermA*	0	0	0	0	1	1	0	0	0	0	0	0
Resistance to fusidic acid	*fusA (p.L461S)*	0	0	1	1	0	0	0	0	0	0	0	0

^1^ Operon; ^2^ Methicillin resistant *S. aureus*; ^3^ 1, presence of the gene; 0, absence of the gene.

**Table 4:** 50% Lethal Time (in days) of *Caenorhabditis elegans* infected by 12 *S. aureus* strains isolated from six patients with PU at D0 and D28. The results are representative of at least three independent trials for each strains. Mantel Cox’s Test was used to compare OP50 or NSA739 with the 12 strains, and each strain between D0 and D28. Wilcoxon Mann Whitney Test was performed to compare LT50 of strains isolated from wounds with improvement versus strains isolated from worsening wounds.

Strains	Clinical Evolution	LT50 (Days)	*p* *(OP 50/Px)*	*p* *(NSA 739/Px)*	*p* *(D0/D28)*
OP50 ^1^	Controls	5.3 (±0.6)	NA	<0.001	NA
NSA739 ^2^		3.0 (±0.2)	<0.001	NA	NA
P5 D0	Improvement	4.0 (±0.1)	<0.001	<0.01	<0.001
P5 D28		4.7 (±0.4)	<0.001	<0.001	
P32 D0	Improvement	3.3 (±0.3)	<0.001	0.301 (NS)	0.003
P32 D28		3.7 (±0.6)	<0.001	<0.001	
P41 D0	Improvement	4.0 (±0.3)	<0.001	<0.001	<0.001
P41 D28		4.8 (±0.7)	<0.001	<0.001	
P7 D0	Worsening	3.7 (±0.7)	<0.001	<0.001	<0.001
P7 D28		4.3 (±0.5)	<0.001	<0.001	
P37 D0	Worsening	4.0 (±0.1)	<0.001	<0.001	<0.001
P37 D28		4.7 (±0.7)	0.001	<0.001	
P50 D0	Worsening	4.6 (±0.2)	<0.001	<0.001	0.207 (NS)
P50 D28		5.0 (±0.1)	<0.001	<0.001	

^1^*E. coli* OP50 strain (low virulent strain); ^2^ NSA739 strain (virulent strain) are tested as control; NS, not significant; NA, not applicable.

## Data Availability

All the identified genomics sequences have been deposited on GenBank website accession bioproject: PRJNA736026.

## Supplementary Materials

The following are available online at https://www.mdpi.com/article/10.3390/genes12121883/s1, Figure S1: Flow chart of the ESCAFLOR Study, Figure S2: Genome-wide representation of the 12 studied *S. aureus* strains isolated from pressure ulcers at D0 and D28, Table S1: Summary of the Whole Genome Sequencing bio-informatic analysis, Table S2: Growth profiles of *S. aureus* strains isolated from pressure ulcers; Ym (maximum absorbance), Y0 (initial absorbance), K (lag time, 1/h), 1/K (inflection point, h), and the extrapolated linear equation of the exponential phase.
