# Supplementary material for: The Persistence of Staphylococcus aureus in Pressure Ulcers: A Colonising Role
Source: Genes (Basel). 2021 Nov 25;12(12):1883. doi: 10.3390/genes12121883 (PMC8701790; doi:10.3390/genes12121883)
Supplement: Supplementary file 1 [file genes-12-01883-s001.zip › genes-1455205-supplementary.pdf]

Supplementary data

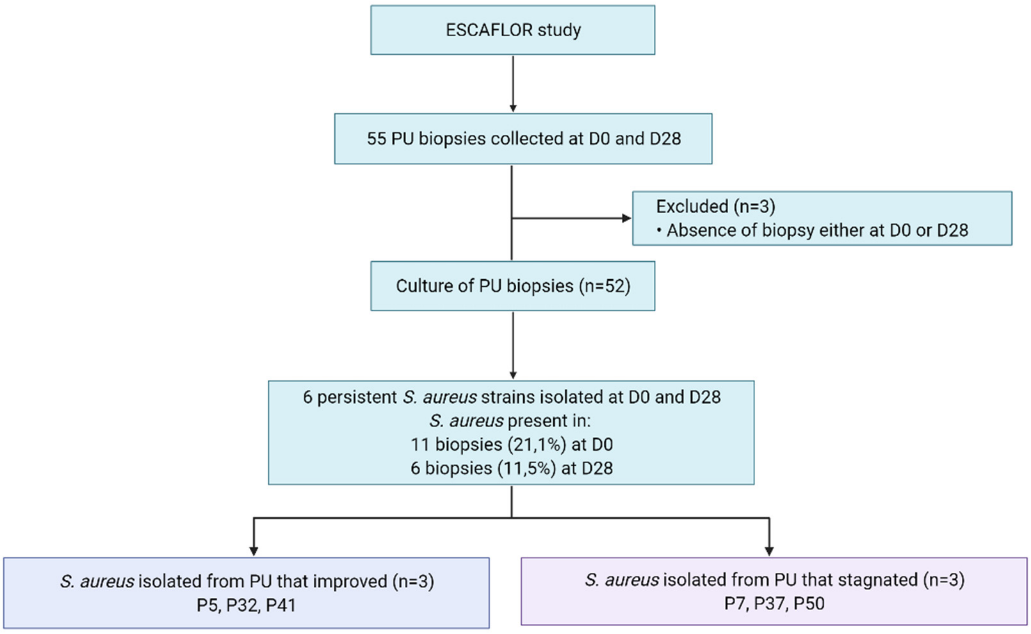

Figure S1: Flow chart of the ESCAFLOR Study.

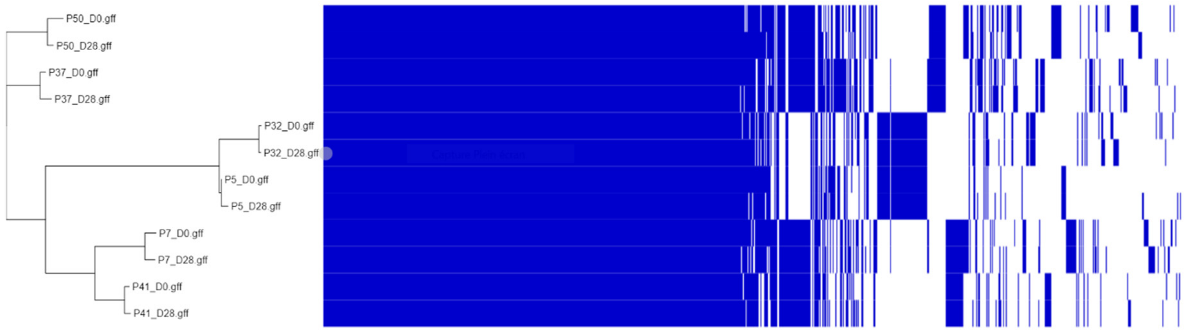

Figure S2: Genome-wide representation of the 12 studied *S. aureus* strains isolated from pressure ulcers at D0 and D28.

Table S1: Summary of the Whole Genome Sequencing bio-informatic analysis.

Feature	Genome size (bp)	Number contigs	G+C Content (%)	Number CDS	Reference sequence used for consensus (GenBank Accession Number)	GenBank Accession Number
P5 D0	2,709,192	75	32.81	2585	NC_017673.1	CP076568
P5 D28	2,710,927	69	32.81	2585		
P7 D0	2,800,844	68	32.62	2710	CP007657.1	CP076719-CP076720
P7 D28	2,800,339	78	32.62	2707		
P32 D0	2,676,228	63	32.78	2524	NZ_CP040230.2	CP076567
P32 D28	2,679,349	69	32.77	2533		
P37 D0	2,754,646	42	32.73	2625	CP007657.1	CP077199-CP077200
P37 D28	2,754,500	46	32.73	2625		
P41 D0	2,695,301	102	32.66	2540	CP026068.1	CP076565
P41 D28	2,695,171	116	32.66	2560		
P50 D0	2,816,720	67	32.75	2732	CP030547.1	CP076564
P50 D28	2,803,529	63	32.75	2704		

Table S2: Growth profiles of *S. aureus* strains isolated from pressures ulcers at D0 and D28.
Ym (maximum absorbance), Y0 (initial absorbance), K (lag time, h-1), 1/K (inflection point, h), and the extrapolated linear equation of the exponential phase.

	Y0	Ym	K (h^{-1})	1/K (h)	Y = ax + b (r^2)
P5 D0	0.141 (0.104 ; 0.181)	1.414 (1.388 ; 1.440)	0.215 (0.193 ; 0.240)	4.648 (4.172 ; 5.175)	y = 0.106x + 0.115 (1)
P5 D28	0.135 (0.100 ; 0.172)	1.383 (1.359 ;1.409)	0.214 (0.193 ; 0.237)	4.675 (4.212 ; 5.186)	y = 0.104x + 0.106 (1)
P7 D0	0.133 (0.099 ; 0.171)	1.390 (1.357 ; 1.426)	0.165 (0.147 ; 0.184)	6.071 (5.434 ; 6.792)	y = 0.082x + 0.087 (1)
P7 D28	0.148 (0.100 ; 0.200)	1.401 (1.359 ; 1.446)	0.175 (0.150 ; 0.203)	5.732 (4.925 ; 6.676)	y = 0.086x + 0.122 (1)
P32 D0	0.127 (0.096 ; 0.161)	1.302 (1.280 ; 1.325)	0.219 (0.199 ; 0.242)	4.565 (4.140 ; 5.031)	y = 0.099x + 0.103 (1)
P32 D28	0.117 (0.088 ; 0.149)	1.273 (1.252 ; 1.295)	0.219 (0.199 ; 0.241)	4.566 (4.149 ; 5.021)	y = 0.097x + 0.086 (1)
P37 D0	0.128 (0.102 ; 0.156)	1.408 (1.388 ; 1.430)	0.204 (0.188 ; 0.221)	4.905 (4.526 ; 5.314)	y = 0.103x + 0.079 (1)
P37 D28	0.131 (0.105 ; 0.158)	1.404 (1.384 ; 1.425)	0.202 (0.187 ; 0.218)	4.950 (4.581 ; 5.348)	y = 0.101x + 0.085 (1)
P41 D0	0.123 (0.0898 ; 0.160)	1.347 (1.318 ; 1.378)	0.188 (0.168 ; 0.210)	5.321 (4.766 ; 5.941)	y = 0.081x + 0.092 (1)
P41 D28	0.124 (0.084 ; 0.169)	1.383 (1.351 ; 1.418)	0.206 (0.181 ; 0.236)	4.843 (4.245 ; 5.518)	y = 0.102x + 0.077 (1)
P50 D0	0.177 (0.132 ; 0.226)	1.409 (1.372 ; 1.450)	0.174 (0.152 ; 0.199)	5.758 (5.034 ; 6.595)	y = 0.084x + 0.175 (1)
P50 D28	0.196 (0.140 ; 0.257)	1.506 (1.464 ; 1.552)	0.184 (0.158 ; 0.215)	5.422 (4.660 ; 6.314)	y = 0.092x + 0.241 (1)
